# Assessing the efficacy, safety and utility of hybrid closed-loop glucose control compared with standard insulin therapy combined with continuous glucose monitoring in young people (≥16 years) and adults with cystic fibrosis-related diabetes (CL4P-CF study): protocol for an open-label, multicentre, randomised, two-arm and single-period parallel trial

**DOI:** 10.1136/bmjopen-2025-111408

**Published:** 2025-10-29

**Authors:** Nithya Kadiyala, Ruth Coleman, Rama Lakshman, Malgorzata E Wilinska, Amanda Brennan, Alistair Lumb, Richard Ian Gregory Holt, Dawn Lau, Parag Yajnik, Yee S Cheah, Shahideh Safavi, Imogen Felton, Gordon MacGregor, Andrew Clayton, Julia Lawton, David Rankin, Steve Churchill, Amanda Adler, Roman Hovorka, Charlotte K Boughton

**Affiliations:** 1 Institute of Metabolic Science–Metabolic Research Laboratories, University of Cambridge, Cambridge, UK; 2 Diabetes Trials Unit, University of Oxford, Oxford, UK; 3 Manchester Adult Cystic Fibrosis Centre, Manchester University NHS Foundation Trust, Manchester, UK; 4 Oxford University Hospitals NHS Foundation Trust, Oxford, UK; 5 Human Development and Health Academic Unit, University of Southampton, Southampton, UK; 6 All Wales Adult CF Centre, University Hospital Llandough, Llandough, UK; 7 Bristol Royal Infirmary, Bristol, UK; 8 Diabetes, King’s College Hospital NHS Foundation Trust, London, UK; 9 St Bartholomew’s Hospital, Barts Health NHS Trust, London, UK; 10 Adult Cystic Fibrosis, Royal Brompton Hospital, London, UK; 11 Queen Elizabeth University Hospital, Glasgow, UK; 12 Glenfield Hospital, University Hospitals of Leicester NHS Trust, Leicester, UK; 13 The University of Edinburgh Centre for Population Health Sciences, Edinburgh, UK; 14 Wolfson Diabetes and Endocrine Clinic, Cambridge University Hospitals NHS Foundation Trust, Cambridge, UK; 15 Diabetes, Royal Papworth Hospital, Cambridge, UK

**Keywords:** Cystic fibrosis, General diabetes, Quality of Life

## Abstract

**Introduction:**

Cystic fibrosis-related diabetes (CFRD) is one of the most clinically impactful comorbidities associated with cystic fibrosis (CF). Current recommended management with insulin therapy is challenging due to variable daily insulin requirements and adds to the significant burden of self-management. This study aims to determine if hybrid closed-loop insulin delivery can improve glucose outcomes compared with standard insulin therapy with continuous glucose monitoring (CGM) in young people (≥16 years) and adults with CFRD.

**Methods and analysis:**

This open-label, multicentre, randomised, two-arm, single-period parallel design study aims to randomise 114 young people (≥16 years) and adults with CFRD. Following a 2–3 weeks’ run-in period, during which time participants use a masked CGM, participants with time in target glucose range (3.9–10.0 mmol/L) <80% will be randomised to 26 weeks with hybrid closed-loop insulin delivery or standard insulin therapy with CGM. The primary outcome is the between-group difference in time in target glucose range (3.9–10.0 mmol/L) based on CGM levels during the 26-week study phase. Analyses will be conducted on an intention-to-treat basis. Key secondary outcomes are time above target glucose range (>10.0 mmol/L), mean glucose and HbA1c. Other secondary efficacy outcomes include glucose and insulin metrics, change in forced expiratory volume in 1 s and body mass index. Safety, utility, participant experiences and participant-reported outcome measures will also be evaluated. The trial is funded by the National Institute for Health and Care Research.

**Ethics and dissemination:**

Ethics approval has been obtained from East of England–Cambridge South Research Ethics Committee. Results will be disseminated by peer-reviewed publications and conference presentations, and findings will be shared with people living with CF, healthcare providers and relevant stakeholders.

**Trial registration number:**

NCT05562492.

**STRENGTHS AND LIMITATIONS OF THIS STUDY**

- The study adopts an open-label, multicentre, randomised parallel design.
- The study includes a 26-week follow-up period with equal numbers of study visits between both groups.
- The study includes young people (≥16 years) and adults with cystic fibrosis-related diabetes, including transplant recipients to support generalisability of the findings.
- The comparator therapy reflects the current standard of care and includes insulin injections or non-automated insulin pump therapy with continuous glucose monitoring.
- The study includes both quantitative and qualitative psychosocial evaluation.

## Introduction

 Cystic fibrosis-related diabetes (CFRD) is the most common comorbidity in cystic fibrosis (CF) affecting 15–20% of adolescents and 35–50% of adults.^1 2^ The prevalence of CFRD is increasing, primarily due to improved survival among people with CF .^3^ As individuals with CF live longer, the risk of developing comorbidities such as CFRD also rises. CF transmembrane regulator (CFTR) modulator therapies have significantly transformed CF management by improving lung function and survival of people living with CF. However, there is no clear consensus on their impact on CFRD.^4 5^

The aetiology of CFRD is complex and the mechanisms leading to the development of CFRD are not fully understood. The mechanisms include reduced and delayed insulin secretion, increased insulin resistance and alterations in the regulation of exocrine function. CFRD is associated with increased frequency of pulmonary infections, deterioration in body weight and lung function and a fourfold greater risk of early mortality.^26^,^10^ Among people with CFRD, hyperglycaemia is also associated with an increased risk of death.^11^ Furthermore, with improved survival of people living with CF, long-term microvascular and macrovascular complications associated with diabetes need to be considered.^12^

The recommended management of CFRD is insulin therapy, which is associated with reduced risk of pulmonary infections and improvements in nutritional status and lung function, but adds further to the burden of CF self-management.^13^,^15^ Insulin requirements can be highly variable day to day due to factors such as malabsorption from exocrine pancreatic insufficiency, pulmonary exacerbations, corticosteroid therapy, diet and use of nutritional support, all of which make diabetes management challenging.

There is a need for novel approaches to enable people with CFRD to attain optimal glucose outcomes while minimising any impact on quality of life. Closed-loop systems (also called automated insulin delivery systems) comprise a control algorithm that automatically modulates insulin delivery via an insulin pump in response to continuous glucose monitoring (CGM) measurements. Hybrid closed-loop (HCL) systems are transforming management of type 1 diabetes due to improvements in glycaemic outcomes and reduced burden of diabetes management.^16 17^

The present study will assess whether HCL insulin delivery is safe and effective at improving glycaemic outcomes compared with standard insulin therapy with CGM in people living with CFRD. We will also explore participants’ experiences of using the system and the impact on quality of life in this population.

## Methods and analysis

### Overview

**Figure 1:**
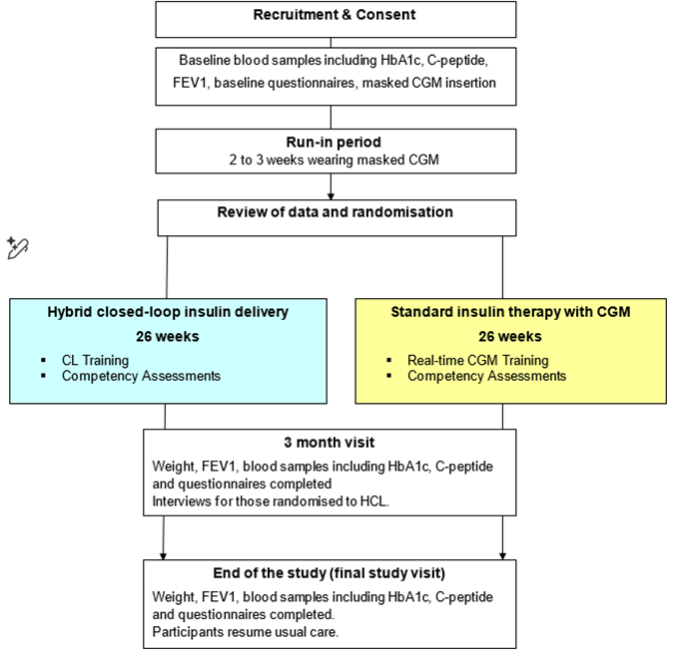
Study flow chart. CGM, continuous glucose monitoring; CL, closed-loop; FEV_1_, forced expiratory volume in 1 s; HCL, hybrid closed-loop.

The study adopts an open-label, multicentre, randomised, two-arm, single-period parallel design to assess the effect of HCL insulin delivery with CGM in young people (≥16 years) and adults with CFRD over 26 weeks compared with standard insulin therapy with CGM over 26 weeks (figure 1). Participants include young people aged 16 years and over and adults with CFRD requiring insulin therapy. The study aims for 114 participants completing the study. Recruitment will target up to 128 participants to allow for withdrawals. After a run-in period, eligible participants will be randomly assigned to 26 weeks of study intervention.

The University of Cambridge (UK) will be the coordinating centre. Clinical sites include:

1. Addenbrooke’s Hospital, Cambridge University Hospital NHS Foundation Trust, UK.
2. Royal Papworth Hospital, Cambridge, UK.
3. Manchester Adult Cystic Fibrosis Centre, Manchester University NHS Foundation Trust, UK.
4. Churchill Hospital, Oxford University Hospital NHS Foundation Trust, UK.
5. Southampton General Hospital, University Hospital Southampton NHS Foundation Trust, UK.
6. All Wales Adult Cystic Fibrosis Centre, University Hospital Llandough, Penarth, Wales, UK.
7. Bristol Royal Infirmary, University Hospitals Bristol NHS Foundation Trust, UK.
8. Kings College Hospital, London, UK.
9. Adult Cystic Fibrosis Service, St Bartholomew’s Hospital, London, UK.
10. Royal Brompton Hospital, London, UK.
11. Queen Elizabeth University Hospital, NHS Greater Glasgow and Clyde, Scotland, UK.
12. Glenfield Hospital, University Hospitals of Leicester, UK.
13. Royal Stoke University Hospital, University Hospitals of North Midlands NHS Trust, UK
14. Belfast City Hospital, Belfast Health and Social Care Trust, Northern Ireland, UK.

Participants may also be recruited from patient identification centres in the East Anglia region for the Addenbrooke’s Hospital site. An interview study exploring participants’ experiences of using closed-loop will be carried out by investigators at the University of Edinburgh.

### Inclusion criteria

1. The participant has CFRD requiring insulin therapy for >3 months.
2. The participant is 16 years of age or older.
3. Baseline time in target glucose range <80% during the run-in period.
4. Forced expiratory volume in 1 s (FEV_1_) >30% of predicted mean for age, sex, race and height at the screening visit or within the past 6 months.
5. Participant is willing to wear/carry study devices 24/7 (CGM/insulin pump/smartphone).
6. Participant is willing to follow study-specific instructions.

### Exclusion criteria

1. Any physical or psychological disease or condition likely to interfere with the normal conduct of the study or interpretation of study results as judged by the investigator.
2. Commencement of CFTR modulator therapy within the previous 1 month.
3. Previous solid organ transplant within the last 12 months or active on transplant waiting list.
4. Use of closed-loop insulin therapy within the past 30 days.
5. Known or suspected allergy to insulin.
6. Severe visual impairment.
7. Severe hearing impairment.
8. Medically documented allergy or unable to tolerate the adhesive of plasters.
9. Serious skin diseases at places of the body corresponding with CGM insertion sites.
10. Participant is pregnant or breastfeeding or planning pregnancy within the next 12 months.

### Study overview

The study includes up to five visits, which can be undertaken in person or remotely with telephone/video link support if preferred, and also includes six telephone/email contacts. Written informed consent will be obtained from all participants before any study-related activities. After a run-in period, participants will be randomised to 26 weeks’ use of HCL insulin delivery or 26 weeks during which they will apply their standard insulin therapy with CGM. Maximum time in the study is 7 months. Table 1 outlines study activities when the participant is randomised to HCL (intervention group). Table 2 outlines study activities when the participant is randomised to standard insulin therapy with CGM (control group).

**Table 1:** Schedule of study visits/contacts when the participant is randomised to closed-loop (intervention group)

	Visit/contact	Description	Start relative to previous/next visit/activity	Duration
Baseline and run-in	Visit 1	Baseline visit: consent, baseline bloods (including HbA1c, C-peptide), height/weight, FEV_1_, urine pregnancy test, questionnaires, masked CGM insertion		1–2 hours
	Visit 2	Review of baseline bloods and CGM data. Randomisation	2–3 weeks after visit 1 (±1 week)	30 min
Postrandomisation training	Visit 3	Study pump, CGM and closed-loop training and initiation, competency assessment	May coincide with visit 2, within 2–4 weeks of visit 1	1–2 hours
CL intervention (26 weeks)	Contact 1	Review use of study devices; study update	Within 48 hours after visit 3	30 min
	Contact 2	Review use of study devices; study update	1 week after visit 3 (±3 days)	30 min
	Contact 3	Review use of study devices; study update; C-peptide measurement	1 month after visit 3 (±2 weeks)	30 min
	Contact 4	Review use of study devices; study update	2 months after visit 3 (±2 weeks)	30 min
	Visit 4	3-month visit FEV_1_, HbA1c, C-peptide, weight, questionnaires, interviews	3 months after visit 3 (±2 weeks)	1 hour
	Contact 5	Review use of study devices; study update; C-peptide measurement	4 months after visit 3 (±2 weeks)	30 min
	Contact 6	Review use of study devices; study update	5 months after visit 3 (±2 weeks)	30 min
	Visit 5	End of closed-loop treatment arm; FEV_1_, HbA1c, C-peptide, weight, questionnaires; resume usual care	6 months after visit 3 (±2 weeks)	1–2 hours

CGM, continuous glucose monitoring; CL, closed-loop; FEV_1_, forced expiratory volume in 1 s.

**Table 2:** Schedule of study visits/contact when the participant is randomised to standard insulin therapy+CGM (control group)

	Visit/contact	Description	Start relative to previous/next visit/activity	Duration
Baseline and run-in	Visit 1	Baseline visit: consent, baseline bloods (including HbA1c, C-peptide), height/weight, FEV_1_, urine pregnancy test, questionnaires, masked CGM insertion		1–2 hours
	Visit 2	Review of baseline bloods and CGM data. Randomisation	2–3 weeks after visit 1 (±1 week)	30 min
Postrandomisation training	Visit 3	CGM training and initiation, competency assessment	May coincide with visit 2, within 2–4 weeks of visit 1	1–2 hours
Standard insulin+CGM (control) (26 weeks)	Contact 1	Review use of study devices; study update	Within 48 hours after visit 3	30 min
	Contact 2	Review use of study devices; study update	1 week after visit 3 (±3 days)	30 min
	Contact 3	Review use of study devices; study update; C-peptide measurement	1 month after visit 3 (±2 weeks)	30 min
	Contact 4	Review use of study devices; study update	2 months after visit 3 (±2 weeks)	30 min
	Visit 4	3-month visit FEV_1_, HbA1c, C-peptide, weight, questionnaires	3 months after visit 3 (±2 weeks)	1 hour
	Contact 5	Review use of study devices; study update; C-peptide measurement	4 months after visit 3 (±2 weeks)	30 min
	Contact 6	Review use of study devices; study update	5 months after visit 3 (±2 weeks)	30 min
	Visit 5	End of standard insulin+CGM treatment arm; FEV_1_, HbA1c, C-peptide, weight, questionnaires; resume usual care	6 months after visit 3 (±2 weeks)	1–2 hours

CGM, continuous glucose monitoring; FEV_1_, forced expiratory volume in 1 s.

### Baseline visit and run-in

At the baseline visit, demographic and medical history data will be collected; questionnaires will be completed; and weight, height and FEV_1_ will be measured. Blood samples for HbA1c and fasted non-hypoglycaemic C-peptide will be collected. A urine pregnancy test will be performed for all females of childbearing age. A masked CGM will be applied to assess baseline glucose levels. They will then proceed to the run-in period (2–3 weeks) where they will use their usual insulin therapy and wear the masked CGM. Participants already using a CGM will continue to use their existing CGM in addition to the masked study CGM during the run-in period. There will be a minimum of 2 weeks’ run-in period for all participants (end of visit 1 to visit 2).

### Randomisation

At the end of the run-in period (2–3 weeks after visit 1), participants’ CGM data will be reviewed. A minimum of 10 days of data recorded, and less than 80% time in target glucose range, will be needed to proceed with randomisation. Eligible participants will be randomised in a 1:1 ratio to the use of HCL or to standard insulin therapy with CGM for 26 weeks. Randomisation will be done using a centrally administered web-based randomisation sequence with a permuted block design (block sizes 2 and 4) and will be stratified by site, age (≤30 years/>30 years) and baseline time in target glucose range (≤65%/>65%). Participants and investigators will not be masked to the intervention used during each period because of the nature of the interventions.

### Closed-loop group

**Figure 2:**
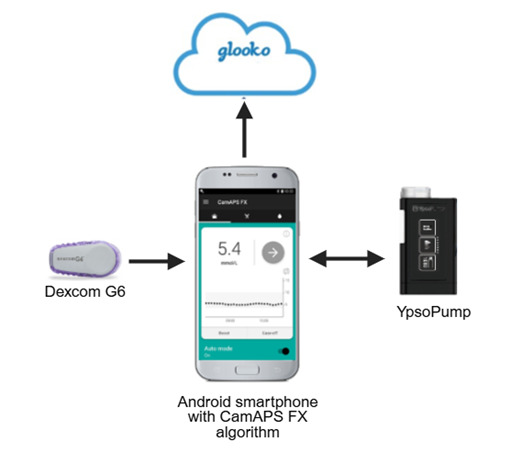
CamAPS FX HCL system. In the present study, we used the CamAPS FX HCL system comprising: YpsoPump insulin pump (Ypsomed, Burgdorf, Switzerland); Dexcom G6 real-time CGM (Dexcom, California, USA) or other approved compatible CGM; smartphone hosting CamAPS FX app with the Cambridge model predictive control algorithm (University of Cambridge, Cambridge, UK) and communicating wirelessly with the insulin pump and CGM transmitter; and cloud upload system to review CGM/insulin data (Glooko, Göteborg, Sweden). The closed-loop algorithm is hosted within the CamAPS FX app which communicates wirelessly with the CGM device and insulin pump. CGM, continuous glucose monitoring; HCL, hybrid closed-loop.

Participants will use the CamAPS FX HCL system (Figure 2) comprising Dexcom G6 CGM (Dexcom, California, USA), YpsoPump insulin pump (Ypsomed, Burgdorf, Switzerland) and the mylife CamAPS FX app (CamDiab, Cambridge, UK) on a standard smartphone. Participants can use their own phone where compatible, or a compatible study smartphone will be provided online supplemental appendix.

Prior to starting HCL insulin therapy, participants will be trained on the use of the study insulin pump, study CGM and CamAPS FX app by the research team. Written guidance will also be provided. Competency on the use of the insulin pump, CGM and HCL system will be assessed. Further training may be delivered as required. Participants will use the HCL system for the next 26 weeks at home.

### Standard insulin therapy with CGM group (control intervention)

Participants will use the Dexcom G6 CGM. Prior to starting the standard insulin therapy with CGM, participants will be trained on the use of the CGM. Written guidance will also be provided. Competency on the use of study CGM will be assessed. Participants will continue their standard insulin therapy with the study CGM for the next 26 weeks at home.

### Contacts during 26-week study period

All participants will be contacted by email or telephone within 48 hours and at 1 week after initiation of the respective study arm. The purpose of this contact is to review the glucose and insulin data, troubleshoot any issues with the devices and record any adverse events or device deficiencies. Thereafter, participants will be followed up through study contacts (telephone/email) at monthly intervals to review glucose data, record any adverse events, device deficiencies, changes in insulin doses, other medical conditions and/or medication. Fasting (non-hypoglycaemic) C-peptide will be measured by dried blood spot completed at home at contact 3 and contact 5.

### Visits during the 26-week home study period

Study visits will occur at 3 and 6 months following initiation of the respective study arm. These study visits will take place at the clinical research facility, outpatient department or remotely if preferred. The purpose of these visits is to review glucose data; to record any adverse events, device deficiencies and changes in insulin doses; and to document other medical conditions and changes in medications. Additionally, a participant’s body weight, height and FEV_1_ will be recorded. Participants will be asked to complete questionnaires to assess quality of life and diabetes management. Participants will have a blood test for HbA1c and complete a fasting (non-hypoglycaemic) C-peptide dried blood spot. At the end of the final study visit, study devices will be collected, and participants will resume their usual diabetes care.

Throughout the trial, clinical care will be provided by the local diabetes/CF teams as per usual care. Participants and/or the clinical team are free to adjust insulin therapy as per usual clinical practice, but no active treatment optimisation will be undertaken by the study team.

### Participant withdrawal criteria

A participant may terminate participation in the study at any time without giving a reason and without any personal disadvantage.

The following prerandomisation withdrawal criteria will apply:

- Participant time in range of >80% during the baseline masked CGM period.
- The participant is unable to demonstrate safe use of the CGM as judged by the investigator.

An investigator can stop the participation of a subject after consideration of the benefit/risk ratio. Possible prerandomisation and postrandomisation withdrawal criteria include:

- Participant is unable to demonstrate safe use of study CGM and/or insulin pump as judged by the investigator.
- Significant protocol violation or non-compliance.
- Recurrent severe hypoglycaemia events related to use of the closed-loop system.
- Recurrent severe persistent hyperglycaemia unrelated to infusion site failure and related to use of the closed-loop system.
- Decision by the investigator or sponsor that termination is in the participant’s best medical interest.
- Allergic reaction to insulin.
- Severe allergic reaction to adhesive surface of infusion set or CGM.
- Serious adverse events.
- Pregnancy, planned pregnancy or breastfeeding.
- Technical grounds (eg, participant relocates).

## Study procedures

### Height and weight

Height and weight will be measured at the baseline visit, 3-month visit and end of study visit. Height will be measured in centimetres using a calibrated measuring device and weight will be measured in kilograms using a calibrated electronic scale.

### Forced expiratory volume in 1 s

Prebronchodilator FEV_1_ will be measured at the baseline visit, 3-month visit and end of study visit using an approved spirometer. The FEV_1_ for eligibility can be recorded within the past 6 months for the baseline visit. Measurements of FEV_1_ for the baseline visit, 3-month visit and end of study visit will be collected within 7 days of the visit using an approved spirometer.

### Blood samples

#### HbA1c

Blood samples for the measurement of HbA1c levels will be taken at the baseline visit, at 3 months and at the final study visit. HbA1c will be measured at a local laboratory using an International Federation of Clinical Chemistry and Laboratory Medicine-aligned method. HbA1c testing will follow the National Glycohemoglobin Standardization Program standards. Blood samples will be disposed of after analysis.

#### C-peptide

Capillary dried blood spot samples for the measurement of fasting (non-hypoglycaemic) C-peptide will be collected at baseline, at 1 month, 3 months, 4 months after randomisation and at the final study visit. Died blood spot samples for C-peptide will be stored deep frozen (−20°C or below) locally until analysis at a central laboratory (Core Biochemical Assay Laboratory, Cambridge, UK). Blood samples will be disposed of after analysis.

#### Glucose

Capillary blood glucose level will be measured just before the C-peptide blood sample is collected to exclude hypoglycaemia (<3.9 mmol/L).

### Masked CGM

During the run-in period, participants will wear a masked Dexcom G6 to collect baseline CGM data and to determine eligibility for randomisation.

### Psychosocial assessments

#### Questionnaires

Surveys used in this trial are listed in table 3. Surveys will be completed at the baseline visit, 3-month visit and end of study visit. Responses will be evaluated at the end of the study once all participants have completed the final study visit.

**Table 3:** Human factors assessment

Questionnaire	Construct measured/relevant points	Timepoint
Problem Areas in Diabetes (PAID) Survey	20-item survey measuring diabetes-related emotional distress and covering a range of negative emotional problems of patients with diabetes.	Baseline, 3 and 6 months
Hypoglycaemia Confidence Survey	Includes 8 different common situations where hypoglycaemia occurs (eg, physical activity, driving) and evaluates the level of confidence in managing it in those situations (2 min).	Baseline, 3 and 6 months
INSPIRE Questionnaire	Survey developed for adults considering or actively using closed-loop. The 31 items cut across quality of life, benefits and burdens of using closed-loop (5 min).	Baseline, 3 months (closed-loop group only) and 6 months (closed-loop group only)
EQ-5D-3L	Developed to describe and value health across a wide range of disease areas. The survey consists of two pages: the EQ-5D descriptive system assessing 5 dimensions of health and the EQ-5D visual analogue scale (2 min).	Baseline, 3 and 6 months
Cystic Fibrosis Questionnaire-Revised (CFQ-R)	Disease-specific survey designed to measure the impact of cystic fibrosis on overall health, daily life, perceived well-being and symptoms. Developed specifically for use in people with a diagnosis of cystic fibrosis. It includes 50 items covering 9 quality of life domains (10 min).	Baseline, 3 and 6 months
Closed-loop experience questionnaire	Feedback questionnaire on closed-loop-specific experience, completed by participants being randomised to the closed-loop intervention arm (2 min).	6 months (closed-loop group only)

INSPIRE, Insulin Dosing Systems: Perceptions, Ideas, Reflections and Expectations Questionnaires-Adults .

#### Qualitative interviews

The qualitative evaluation will adopt a cross-sectional design in which (n=16–20) participants in the HCL arm will be interviewed once after they have at least 3 months’ experience of using the HCL system. Purposive sampling will be used to ensure diversity in terms of age, gender, CFRD duration and sociodemographic variables such as education, ethnicity and occupation. Interviews will begin by exploring participants’ lives and experiences of self-managing diabetes before using closed-loop, with a particular focus on the distinctive issues and challenges resulting from living with both CF and CFRD. The interview will then be used to explore whether and how using closed-loop has affected participants’ CFRD management experiences and everyday (work/school/family) lives.

### Patient and public involvement

Patients and the public were involved in the research from the very beginning in the development of this protocol. We worked in collaboration with the Cystic Fibrosis Trust to form a patient and public involvement (PPI) group which included people living with CFRD and relatives/caregivers of people living with CFRD. This group endorsed the primary endpoint (time in target glucose range) as being patient relevant and highlighted that research into interventions to reduce treatment burden was the top priority in the James Lind Alliance Priority Setting Partnership for CF at the time. The inclusion of the qualitative components to the trial was also endorsed by the PPI group. The PPI group has also been involved in other aspects of the trial design, including reviewing participant-facing study documents, inclusion and exclusion criteria and patient-relevant outcomes. One of the coapplicants of the study is a lay representative living with CFRD who has been involved in the study design from the outset. A person living with CFRD is a member of the Trial Steering Committee (TSC).

### Statistical analysis

All randomised participants with/without protocol violation and including dropouts and withdrawals will be included in the analysis according to intention-to-treat principle.

#### Primary outcome analysis

The primary analysis will evaluate the between-group difference in the proportion of time spent in the target glucose range of 3.9 and 10.0 mmol/L based on CGM levels during the 26-week intervention period.

The values will be compared using a linear mixed model; the dependent variable is the primary endpoint and the independent variable is treatment allocation, adjusting for baseline time in target glucose range, age and clinical centre. A 95% CI will be reported for the difference between the interventions based on the linear mixed model. Residual values will be examined for an approximate normal distribution. If values are highly skewed, then a transformation or robust statistical method will be used instead. A 5% significance level will be used to declare statistical significance for the primary comparison. A two-sided p value will be reported. The primary analysis will be a single statistical comparison of a single outcome measure.

#### Key secondary endpoints

Analysis of secondary endpoints will parallel the primary analysis. A transformation will be applied to all highly skewed secondary endpoints.

The following key and secondary endpoints will be assessed for between-group difference:

- Proportion of time with sensor glucose >10.0 mmol/L (%) throughout treatment period.
- Mean sensor glucose (mmol/L) throughout treatment period.
- HbA1c (mmol/mol) at end of treatment period.

#### Other secondary efficacy endpoints

##### Glycaemic endpoints (throughout the treatment period)

- Proportion of time with sensor glucose <3.9 mmol/L (%).
- Proportion of time with sensor glucose <3.0 mmol/L (%).
- Proportion of time with sensor glucose >13.9 mmol/L (%).
- Proportion of time with sensor glucose in tight range of 3.5–7.8 mmol/L (%).
- SD (mmol/L) and coefficient of variation (%) of glucose to quantify the glucose variability.

##### Insulin endpoints (throughout the treatment period)

- Total daily insulin dose (units/day).
- Total daily basal insulin dose (units/day).
- Total daily bolus insulin dose (units/day).

Trends in sensor glucose and insulin data collected within intervention arms will be evaluated on a monthly basis, and daytime (06:00 to 23:59) and overnight (00:00 to 05:59) glucose control will be evaluated separately.

##### Clinical endpoints (throughout the treatment period)

- Change in fasting C-peptide (pmol/L).
- Change in body mass index (kg/m^2^).
- Change in percentage of predicted FEV_1_.
- Number of pulmonary exacerbations.
- Number of hospitalisations.

The Hochberg method will be used to adjust for multiple testing of secondary endpoints.

### Utility analysis

Over the 26-week period, the proportion of time with sensor glucose will be tabulated for each treatment arm, in addition to the proportion of time with closed-loop system active in the closed-loop arm.

### Safety analysis

For each of the following safety outcomes, mean±SD or summary statistics appropriate to the distribution will be tabulated by treatment group:

- Number of severe hypoglycaemia events.
- Number of participants with any severe hypoglycaemia event.
- Number of adverse events per participant.
- Number of serious adverse events per participant.

All of the above safety outcomes will be tabulated for all participants (including dropouts and withdrawals), regardless of whether CGM data are available and irrespective of whether closedloop was operational. All serious adverse events will be listed for the entire study duration.

### Human factor evaluation

#### Questionnaires

Descriptive tabulations of questionnaires will be carried out, and scores will be calculated using the provided scaling and scoring tools as appropriate. The between-group difference of each score will be assessed using a linear mixed model, adjusting for the corresponding score at baseline, age and clinical centre.

#### Qualitative interviews

Data will be analysed thematically using the constant comparison method. NVivo, a qualitative software package, will be used to facilitate data coding/retrieval.

### Per-protocol analysis

A per-protocol analysis restricted to participants with a minimum of 60% CGM data during the control period and 60% use of closed-loop during the closed-loop period will be conducted for the primary endpoint.

### Interim analysis

No formal interim analysis will be performed. Interim analyses of the safety data will be performed at regular intervals (at least six monthly) for review by the Data Monitoring and Ethics Committee (DMEC).

### Sample size calculation

The primary analysis will evaluate the between-group difference in the proportion of time in target glucose range of 3.9 and 10.0 mmol/L based on sensor glucose from initiation of the treatment arm to 26 weeks. Data from four studies were considered when preparing the sample size calculations for this study.^18^,^21^ For a clinically important 5.0 percentage point absolute mean difference in time with glucose in target range,^22^ an SD of 8.1, 90% power, two-sided t-test at 5% significance level and 1:1 randomisation, total sample size is estimated to be 114 participants (57 per treatment group). Allowing for 10% loss to follow-up (5–8% in previous studies), we aim to randomise a total of 128 participants (64 per treatment group).

## Study management

### Study sponsor

The study sponsors are the Cambridge University Hospitals NHS Foundation Trust and the University of Cambridge.

### Data Monitoring and Ethics Committee

A DMEC will comprise an independent chair and two experts. The DMEC aims to safeguard the interests of trial participants, assess the safety of the interventions during the trial and monitor the overall conduct of the clinical trial.

The DMEC will receive and review the progress and accruing data of the clinical trial and provide advice on the conduct of the trial. The DMEC will be informed of any related serious adverse events and any unanticipated adverse device effects that occur during the study and will review compiled adverse event data at periodic intervals.

### Trial Steering Committee

A TSC will include an independent chair, two experts, a PPI member and the chief investigator. This committee will supervise the trial, to ensure it is conducted to high standards in accordance with the protocol, the principles of Good Clinical Practice (GCP) and with regard to participant safety.

The TSC will meet at regular intervals during the active phase and at the conclusion of the study. The TSC will consider the study and relevant information from other sources, ensuring at all times that ethical considerations are met when recommending the continuation of the trial.

### Trial Management Group

The Trial Management Group (TMG) will meet fortnightly and will be responsible for day-to-day management of the trial. The TMG will consist of the chief investigator, study coordinators and the data manager.

### Data management and monitoring

The study coordinators and data manager will be responsible for maintaining quality assurance and quality control systems to ensure that the trial is conducted and data are generated, documented and reported in compliance with the protocol, GCP and ethics requirements.

Confidentiality of participant data shall be observed at all times. Personal details for each participant taking part with a link to a unique identification number will be held locally in the Trial Site File at each study site. Electronic case report forms will be used for recording anonymised study data and will be completed in accordance with GCP and ISO 14155:2020 guidelines. All results will remain anonymous.

### Indemnity

The clinical investigators are indemnified to cover negligent harm to patients participating in the study by their membership of medical defence organisations. National Health Service indemnity cover will apply for any claims arising from management and conduct of research. Any liability arising from study design will be covered by the clinical trial insurance policy organised by the University of Cambridge.

## Ethics and dissemination

The study has received approval from the East of England–Cambridge South Research Ethics Committee (UK) (22/EE/0164). All participants will be provided with verbal and written information about the trial and procedures involved in the study before providing written informed consent.

Standard operating procedures for monitoring and reporting of all adverse events and adverse device effects will be in place, including serious adverse events, serious adverse device effects and specific adverse events such as severe hypoglycaemia and significant hyperglycaemia with ketosis.

Any substantial amendments to the protocol and other documents shall be notified to and approved by the funder and the Research Ethics Committee prior to implementation as per nationally agreed guidelines.

Screening and recruitment commenced in February 2023, and the study is expected to be completed by August 2026. Study results will be disseminated through peer-reviewed publications, conference presentations and lay communications.

## Supplementary material

10.1136/bmjopen-2025-111408 online supplemental file 1
