# Supplementary material for: Assessing the efficacy, safety and utility of hybrid closed-loop glucose control compared with standard insulin therapy combined with continuous glucose monitoring in young people (≥16 years) and adults with cystic fibrosis-related diabetes (CL4P-CF study): protocol for an open-label, multicentre, randomised, two-arm and single-period parallel trial
Source: BMJ Open. 2025 Oct 29;15(10):e111408. doi: 10.1136/bmjopen-2025-111408 (PMC12574440; doi:10.1136/bmjopen-2025-111408)

**Supplementary Appendix**

**Table of Contents**

[1. Training details 2](#_Toc209607206)

[2. Example consent form 4](#_Toc209607207)

# Training details

*Closed-loop training*

*Training on the use of the study insulin pump*

This will cover key aspects of insulin pump use and particular attention will be paid to

- Insulin cartridge and infusion set changes and correct priming procedure
- Stopping and starting insulin delivery via the insulin pump

*Training on the use of the study glucose sensor*

This will cover key aspects of the study glucose sensor and particular attention will be paid to

- Insertion and initiation of sensor session
- Interpreting real-time sensor data and trend arrows
- Setting and adjustment of alarms/alerts

*Training on the use of the closed-loop system*

This will cover key aspects of the closed-loop app and particular attention will be paid to

- Use of the bolus calculator in the app
- Managing hypo- and hyperglycaemia
- Managing exercise/activity
- Sick day rules

Written, easy to use guidance for the operation of the insulin pump, glucose sensor and closed-loop system will also be provided.

*Standard therapy*

*Training on the use of the study glucose sensor*

This will cover key aspects of the study glucose sensor device and particular attention will be paid to

- Insertion and initiation of sensor session
- Interpreting real-time sensor data and trend arrows
- Setting and adjustment of alarms/alerts
- Managing hypo- and hyperglycaemia

Written, easy to use guidance for the operation of glucose sensor will be provided.

#
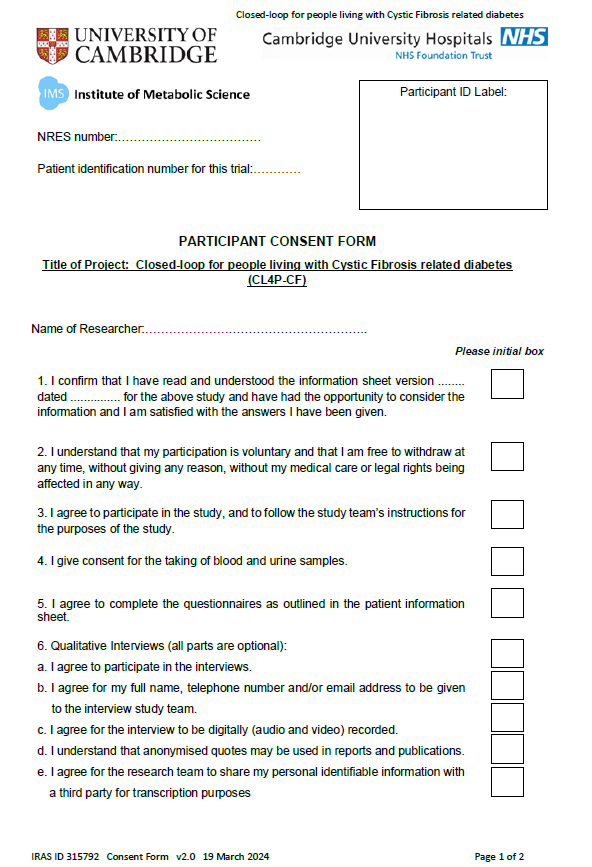
Example consent form


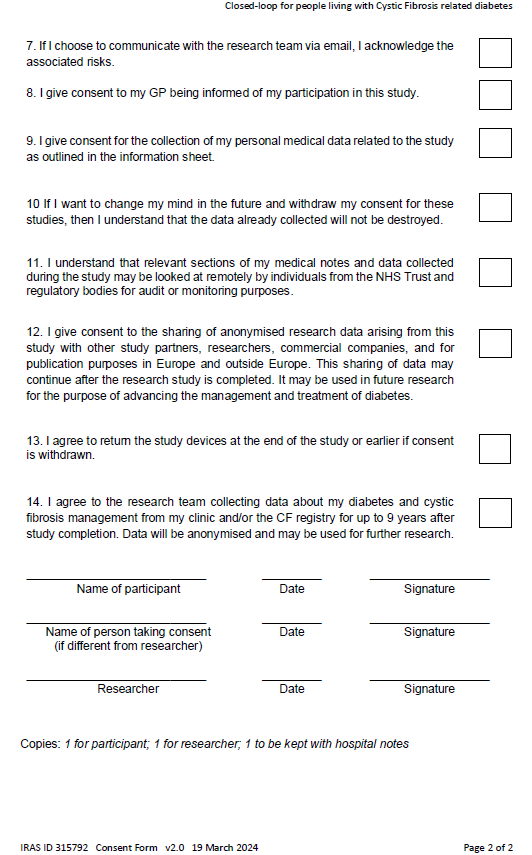

Supplement: online supplemental file 1 [file bmjopen-15-10-s001.docx]
