# Supplementary material for: Assessing the efficacy, safety and utility of hybrid closed-loop glucose control compared with standard insulin therapy combined with continuous glucose monitoring in young people (≥16 years) and adults with cystic fibrosis-related diabetes (CL4P-CF study): protocol for an open-label, multicentre, randomised, two-arm and single-period parallel trial
Source: BMJ Open. 2025 Oct 29;15(10):e111408. doi: 10.1136/bmjopen-2025-111408 (PMC12574440; doi:10.1136/bmjopen-2025-111408)
Supplement: Supplementary file 2 [file bmjopen-2025-111408.reviewer_comments.pdf]

PEER REVIEW HISTORY

BMJ Open publishes all reviews undertaken for accepted manuscripts. Reviewers are asked to complete a checklist review form and are provided with free text boxes to elaborate on their assessment. These free text comments are reproduced below.

ARTICLE DETAILS

Title (Provisional)

Assessing the efficacy, safety and utility of hybrid closed-loop glucose control compared to standard insulin therapy combined with continuous glucose monitoring in young people (≥16 years) and adults with cystic fibrosis related diabetes (CL4P-CF study): protocol for an open-label, multi-centre, randomised, two arm and single period parallel trial

Authors

Kadiyala, Nithya; Coleman, R; Lakshman, Rama; Wilinska, Malgorzata E; Brennan, Amanda; Lumb, Alistair; Holt, Richard Ian Gregory; Lau, Dawn; Yajnik, Parag; Cheah, Yee S; Safavi, Shahideh; Felton, Imogen; MacGregor, Gorgdon; Clayton, Andrew; Lawton, Julia; Rankin, David; Churchill, Steve; Adler, Amanda I; Hovorka, Roman; Boughton, Charlotte K

This article was not externally reviewed at BMJ Open. Protocols that have been independently assessed prior to submission to BMJ Open are usually fast-tracked to publication on the grounds that further substantial changes will not be possible. This independent assessment will usually be external, independent review for both a substantial grant award from a non-commercial or government funder and ethics approval.
